# Impact of grassland saline-alkaline degradation on domestic herbivore rumen microbiota and methane emissions

**DOI:** 10.3389/fvets.2025.1598973

**Published:** 2025-07-15

**Authors:** Yizhen Wang, Xin Jiang, Guangming Ma, Youran Sun, Xue Wang, Haixia Sun, Yanan Li, Ling Wang

**Affiliations:** ^1^Key Laboratory of Vegetation Ecology of the Ministry of Education, Jilin Songnen Grassland Ecosystem National Observation and Research Station, Institute of Grassland Science, Northeast Normal University, Changchun, China; ^2^Northeast Institute of Geography and Agroecology, Chinese Academy of Sciences, Harbin, China

**Keywords:** grassland degradation, global warming, herbivore grazing, methane emissions, rumen microbes

## Abstract

**Introduction:**

Grazing ruminant production has the risk of degrading the environment beyond natural recovery due to their production of enteric methane (CH_4_) which is the main contributor to the increase in global CH4 emissions. In particular, grasslands are currently experiencing severe saline-alkaline degradation that is prevalent in arid and semi-arid grassland areas globally. Yet, the impact of grassland saline-alkaline degradation-induced alterations in plant resources on herbivore, and subsequent CH4 emissions, remain underexplored.

**Methods:**

Here we examined these effects by feeding domestic ruminant-sheep with plants from undegraded (UG), moderately degraded (MG), and severely degraded grasslands (SG), focusing on rumen key microbes and nutrition process.

**Results:**

Our results showed that moderately and severely saline-alkaline degradation of grasslands differently influences rumen key microbes associated with CH_4_ synthesis, thereby affecting CH_4_ emissions of ruminants. Specifically, the relative abundance of *Treproema* that can competitively inhibit the CH_4_ production was significantly increased in MG-fed sheep, which resulted in reduced CH_4_ emissions. Conversely, the relative abundance of *Methanosphaera* that positively related to CH_4_ production was significantly increased in SG-fed sheep, which resulted in increased CH_4_ emissions. Forage resources in severely degraded grasslands exhibited extremely high sodium (Na) content, while high forage diversity was found in moderately degraded grassland. Further, we found that increased Na intake has a significant influence on the abundance of *Methanosphaera*.

**Discussion:**

Taken together, our study provides novel insights into the underlying mechanism of the CH_4_ emissions induced by saline-alkaline degradation in ruminant herbivores; the increase in Na intake induced by grassland saline-alkaline degradation could be an important factor affecting rumen Methanosphaera thereby CH_4_ emissions by livestock. Our findings suggest that increasing grassland saline-alkaline degradation worldwide will greatly change the risk of CH_4_ emissions from grazing ruminants depending on the degree of degradation, which should be incorporated into future consideration of grassland carbon budgets.

## 1 Introduction

Livestock production, particularly ruminant production, carries the risk of deteriorating the environment beyond natural recovery (1). This is because the enteric methane (CH_4_) from ruminants is a major contributor to the increase in global CH_4_ emissions, which greatly increases the risk of global warming (2). Notably, ruminants from grazing systems, usually have higher CH_4_ emissions relative to stable-fed ruminants with high intake of nutrient-dense rations (3–5). More importantly, estimates suggest that approximately 50 percent of the grasslands in the world have already shown degradation to some extent as a result of climate change and human activities (6). Particularly saline-alkaline degradation, which is prevalent in arid and semi-arid grassland areas globally (7), is likely to further greatly increase CH_4_ emissions from grazing ruminants. However, the impact of saline-alkaline grassland degradation induced alterations in plant resources on the CH_4_ of grazing ruminants, which are an important component of managed grassland ecosystems (8), still remains poorly understood.

Methanogenesis is a normal process that mostly occurs during the anaerobic fermentation of feed by a microbial consortia in the rumen (9). In the fermentation process, the carbohydrates in the feed are first fermented by rumen fungi and bacterial communities to produce energetic substrates utilized by the host animal, as well as carbon dioxide (CO_2_) and hydrogen (H_2_). Subsequently, the CO_2_ and H_2_ generated are mainly converted to CH_4_ by rumen methanogens, whereas can also be converted to useful metabolites by specific bacteria (10). For the methanogenic pathway, rumen archaea including *Methanobacterium*, *Methanobrevibacter*, *Methanomicrobium*, *Methanoculleus*, *Methanosarcina* and *Methanosphaera* have been demonstrated to exhibit strong CH_4_-producing capacities by converting H_2_ and CO_2_ into CH_4_ (11, 12). Conversely, certain rumen bacterial genera, such as *Ruminococcus*, *Butyribacterium*, *Clostridium* and *Treponema*, have been shown to compete with methanogens for H_2_ and CO_2_, redirecting these substrates toward the synthesis of alternative metabolic products, such as acetate (13). In addition, protozoa populations, as the engineers of rumen microbial ecosystem, are also able to provide H_2_ for methanogens to produce CH_4_ (14). Therefore, the production of CH_4_ is driven by the interaction of methanogens with other rumen microbes, including protozoa, bacteria, and fungi. Evidence from experimental study has demonstrated the interaction process of various rumen microbes was mainly modulated by diet nutrient profiles (15), and thus, saline-alkaline grassland degradation may affect the CH_4_ emissions of grazing ruminants by altering their nutrition intake and further affecting the rumen microbial community. Indeed, a previous study found that the fiber content in grassland plant resources showed a linear decrease with increasing grassland degradation level due to the decrease in perennial plants and increase in annual plants (16, 17). The decrease in fiber intake could reduce the CH_4_ emissions of ruminants by affecting the relative abundance of fungi and bacteria in the rumen to reduce the supply of CO_2_ and H_2_ available to methanogens (18). Further study also found that the fat content in grassland plant resources showed a linear increase with increasing grassland degradation level (19). The increase in fat intake could reduce the CH_4_ emissions of ruminants through competition of unsaturated fatty acids for H_2_ (20, 21). Meanwhile, the decreased calcium content in grassland plant resources induced by saline-alkaline degradation also can result in more CH_4_ emissions, on average, of ruminants via increasing the relative abundance of methanogens in the rumen (22–24). Furthermore, different levels of grassland saline-alkaline degradation induced alterations in plant diversity, salt content and secondary metabolites may also affect the CH_4_ emissions of ruminants. Consequently, the process underlying the effects of grassland saline-alkaline degradation on the CH_4_ emissions from grazing ruminants is extremely complex and unpredictable.

Here, we examined the effects of grassland saline-alkaline degradation on ruminant CH_4_ emissions together with the underlying impact mechanisms by feeding small-tailed sheep diets that simulated forage availability in grasslands with different levels of degradation.

## 2 Materials and methods

### 2.1 Animals, experimental design, and diets

This experiment was conducted at the experimental base of the Institute of Animal Husbandry, Heilongjiang Academy of Agricultural Sciences (45°42′N, 126°38′E). All animal-based experiments were conducted in accordance with the principles and responsibilities outlined in the Northeast Normal University’s (Changchun, China) guidelines for animal research. Twelve male small-tailed Han sheep of similar weight (30.49 ± 4.31 kg, mean ± SD) were selected for this study. They were each assigned to 1 of 3 dietary treatments which represent different degrees of degraded grasslands classified by the biomass of grassland plants (Areas with 40–60% vegetation cover were deemed as moderately degraded, and those with less than 40% vegetation cover were regarded as heavily degraded) (25): (1) undegraded grassland plants (UG); (2) moderately degraded grassland plants (MG); or (3) severely degraded grassland plants (SG). Plant samples from grasslands degraded to different levels were obtained from the Songnen grassland in Jilin, China (44°45′N, 123°45′E) and plants were cut and baled for feeding to sheep. The grasslands in the area have already shown saline-alkaline degradation to different levels as a result of climate change and overgrazing. The climate of the area is continental with average annual temperatures ranging from 2.4°C to 2.7°C and precipitation ranging from 300 mm to 500 mm, most of which occurs between June and August (data from Changling County Climate Station, Jilin Province).

**Figure 1:**
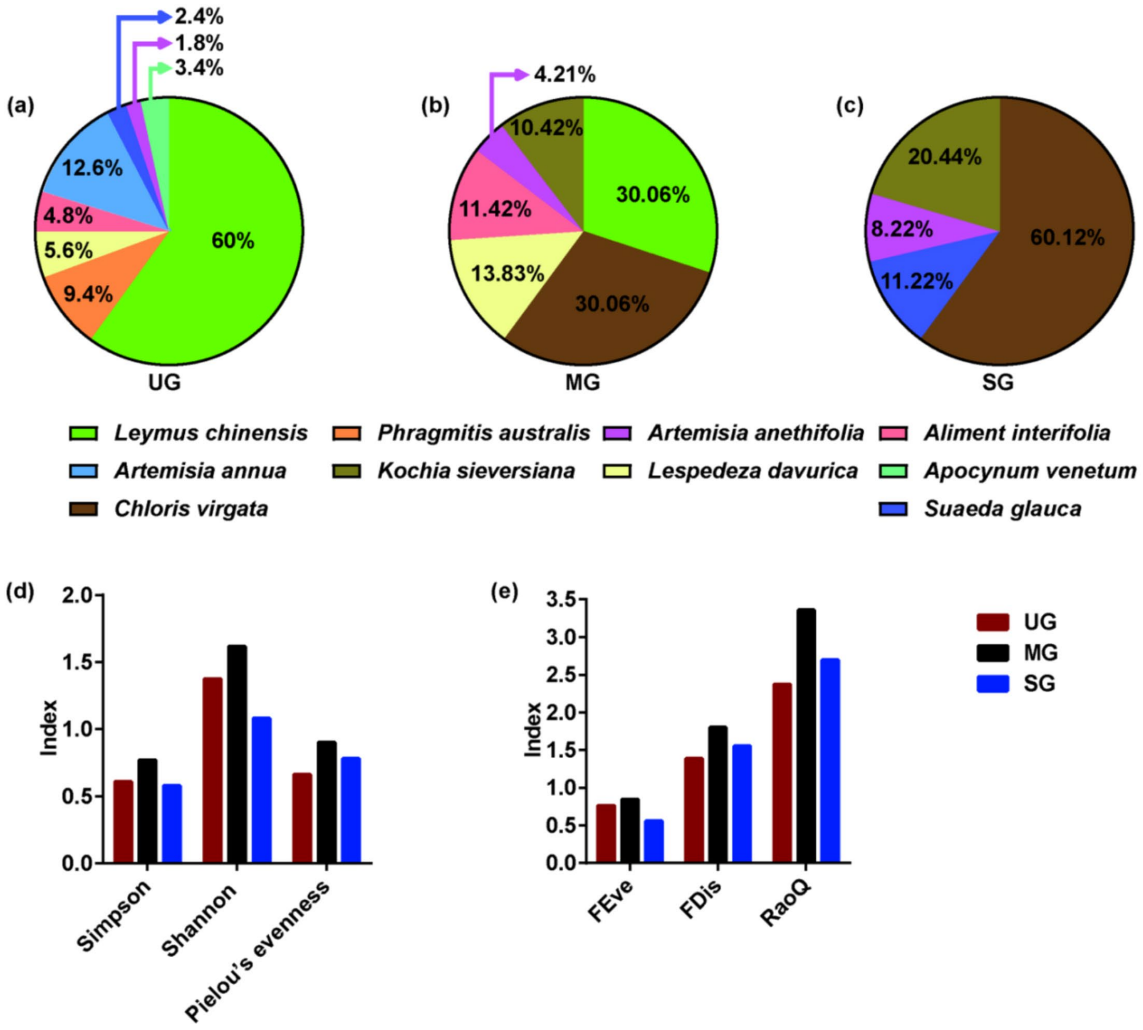
Plant composition **(a–c)**, species diversity **(d)**, and functional diversity **(e)** of grasslands with different levels of saline-alkaline degradation. UG undegraded grassland, MG moderately degraded grassland, SG severely degraded grassland, FEve functional evenness, FDis functional dispersion, RaoQ Rao’s quadratic entropy index.

We established three 400-m parallel transects at 50-m intervals in grasslands with different levels of degradation (UG: undegraded grassland plants, MG: moderately degraded grassland plants, and SG: severely degraded grassland plants). Next, we determined the locations of 100 cm × 100 cm quadrants every 40-m along each transect. We used these quadrants to measure the composition and proportion of plants, and then plant samples were harvested at a cutting height of approximately 10 cm. The diets of the sheep were formulated according to the types and proportions of plants in actual grasslands degraded to different levels, as presented in Figure 1. Throughout the 8-week feeding trial, the sheep were fed individually in cages, which were spacious enough for them to stand and lie down in. The sheep were weighed on the final day of the trial period to determine average daily weight gain (ADG) which calculated by dividing the difference in body weight by the number of days. The sheep were fed twice per day, and allowed to feed and drink *ad libitum* throughout the experiment period.

### 2.2 Sample collection

The feed offered and refused for individual sheep was weighed each day to calculate the daily dry matter intake (DMI). Samples of various plants and formulated diets were collected once a week and later pooled by week. Subsequently, all feed samples were dried at 65°C for 72 h, milled to pass through a 1-mm mesh screen, then stored in sealed bags (150 mm × 220 mm) at 4°C until chemical composition was determined.

Ruminal fluid samples were collected on the final day of the experiment before the morning feeding via an oral stomach tube equipped with a vacuum pump. Approximately 10 mL of ruminal fluid initially collected was discarded to reduce the chance of contamination of the fluid sample in the stomach tube with saliva. After that, 4 layers of cheesecloth were used to strain the ruminal fluid, and the pH was measured immediately. Then, 10 mL of the filtrate is acidified by mixing with 2 mL of metaphosphoric acid (25%, wt/vol) and the mixture was centrifuged at 3,000 × g for 15 min. The supernatant was separated and stored at −20°C until volatile fatty acids (VFA) was determined. Another 4 mL of filtrate was immediately stored in liquid nitrogen until the microbial community was analyzed.

### 2.3 Analysis of the nutritional composition of feeds

Samples of each plant and diets were sent to the Animal Nutrition Laboratory of Northeast Agricultural University (Harbin, China) for nutrient analysis using wet chemistry methods. Contents of the dry matter (DM, method 934.01), ash (method 942.05), ether-extract (EE, method 920.39) and crude protein (CP, method 988.05) in feeds were assayed in accordance with the procedures of AOAC International (26). The neutral detergent fiber (NDF) and acid detergent fiber (ADF) contents were measured based on the method described by previous study (27), which the heat-stable *α*-amylase was used to treat the feeds. The mineral element content was measured using an inductively coupled plasma-optical emission spectrometer (ICP-6800S, Shanghai Meixi Instrument Co., Ltd., China).

### 2.4 Analysis of ruminal fermentation parameters and microbial community

Concentrations of acetate and propionate in the rumen fluid samples were determined using gas chromatography (Shimadzu GC-2010, Japan) (28).

Total DNA in the rumen fluid was extracted using a DNA extraction kit (Shanghai Shengye Biotech, China). The V3 and V4 region of bacteria and archaea was used for 16S rRNA gene sequencing while the fungus was used for 18S rRNA gene sequencing, with all amplicon libraries preparation and sequencing performed on a MiSeq platform (Illumina, San Diego, CA, United States). The fluorescent quantitative polymerase chain reaction (PCR) amplification reaction conditions were pre-denaturation 98°C (30 s); denaturation 98°C (15 s), annealing temperature 50°C (30 s); extension temperature 72°C (30 s), 30 cycles, and final extension 72°C (5 min). After being recycled from a 1.8% agarose gel, the PCR products were purified using an OMEGA DNA purification column (Gene Company Limited). The purified products were quantified using a Quant-iT PicoGreen dsDNA Assay Kit (Gene Company Limited) in accordance with the kit’s instructions. The DNA fragments were paired-end sequenced by an Illumina miseq/novaseq. The DADA2 method was used to perform the steps of de-priming, mass filtering, denoising, splicing, and de-chimerization. Sequencing data were processed using MacQIIME version 1.9.1. Primers and homopolymer runs (maximum length, 8) of the joined sequences were trimmed after paired-end forward and reverse reads were joined. Only sequences ≥400 bp in length, with both an average quality score ≥ 25 and ambiguous bases ≤6 remained for downstream analysis. UCHIME (29) was used for *de novo* chimera checking, and USEARCH (30) was used for operational taxonomic unit (OTU) identification in order to identify similar sequences that had >97% similarity. BLAST (31) was used to assign representative sequences for bacterial and archaeal OTUs to the Greengenes16S rRNA gene database [version gg_13_8; (32)] and RIM-DB database (33), respectively.

### 2.5 *In vitro* rumen fermentation

The *in vitro* fermentation device adopted in this study was the artificial rumen simulating system MC-ABSF-II (Beijing Mancang Technology Co., Ltd., China). The protocol used for the *in vitro* incubation was described by Li et al. (34). Sheep rumen fluid was collected prior to morning feeding, filtered through four layers of cheesecloth, and combined with artificial saliva (39°C) at a 2:1 ratio (buffer: ruminal fluid, v: v) (35). Dispensing the 150 mL of buffered ruminal fluid into 200 mL incubation flasks that had been preheated. In each incubation flask, 2 g of each substrate were mixed with the buffered ruminal fluid. The mixture was then incubated for 24 h at 39°C in a hot water bath shaker. Using a real-time *in vitro* fermentation system (made by Jilin Academy of Agricultural Sciences, model Qtfxy-6), the milliliter (mL) of CH_4_ from each flask was monitored.

### 2.6 Statistical analysis

R Statistical Software (v4.1.2; (36)) was used to analyze all of the experiment’s data. One-way ANOVA (LSD) were used to analyze the effects of grassland degradation on nutrient intake, rumen fermentation parameters and methane emission of sheep. To analyze the effects of nutrient intake to rumen microbial relative abundance, we used linear mixed effects model (LMMs). In this model, nutrient intakes were taken as fixed factors and grassland types (undegraded grassland plants, moderately degraded grassland plants and severely degraded grassland plants) were taken as random factors. Specifically, the equation of model is *Yᵢ_j_* = *X_ᵢj_β* + *Z_ᵢj_μ_i_ + ϵ_ᵢj_*, where *Yᵢ_j_* is the observed response for the *j*-th observation within group *i*, *X_ᵢj_* is a vector of covariates associated with the fixed effects for the *j*-th observation in group *i*, *β* is the population mean which represents fixed-effect factors, *Z_ᵢj_* is a vector of variables associated with the random effects for the *j*-th observation in group *i*, *μ_i_* is intercepts relative to population mean across different groups which means variability between groups, *ϵ_ᵢj_* is the error term for the *j*-th observation in group *i* representing the unexplained variability within the group. Statistically significant differences among treatments were analyzed using Tukey’s test. Significant differences were declared at *p* ≤ 0.05, and marginally significant differences were defined at 0.05 < *p* ≤ 0.10.

## 3 Results

### 3.1 Growth performance and rumen fermentation

**Figure 2:**
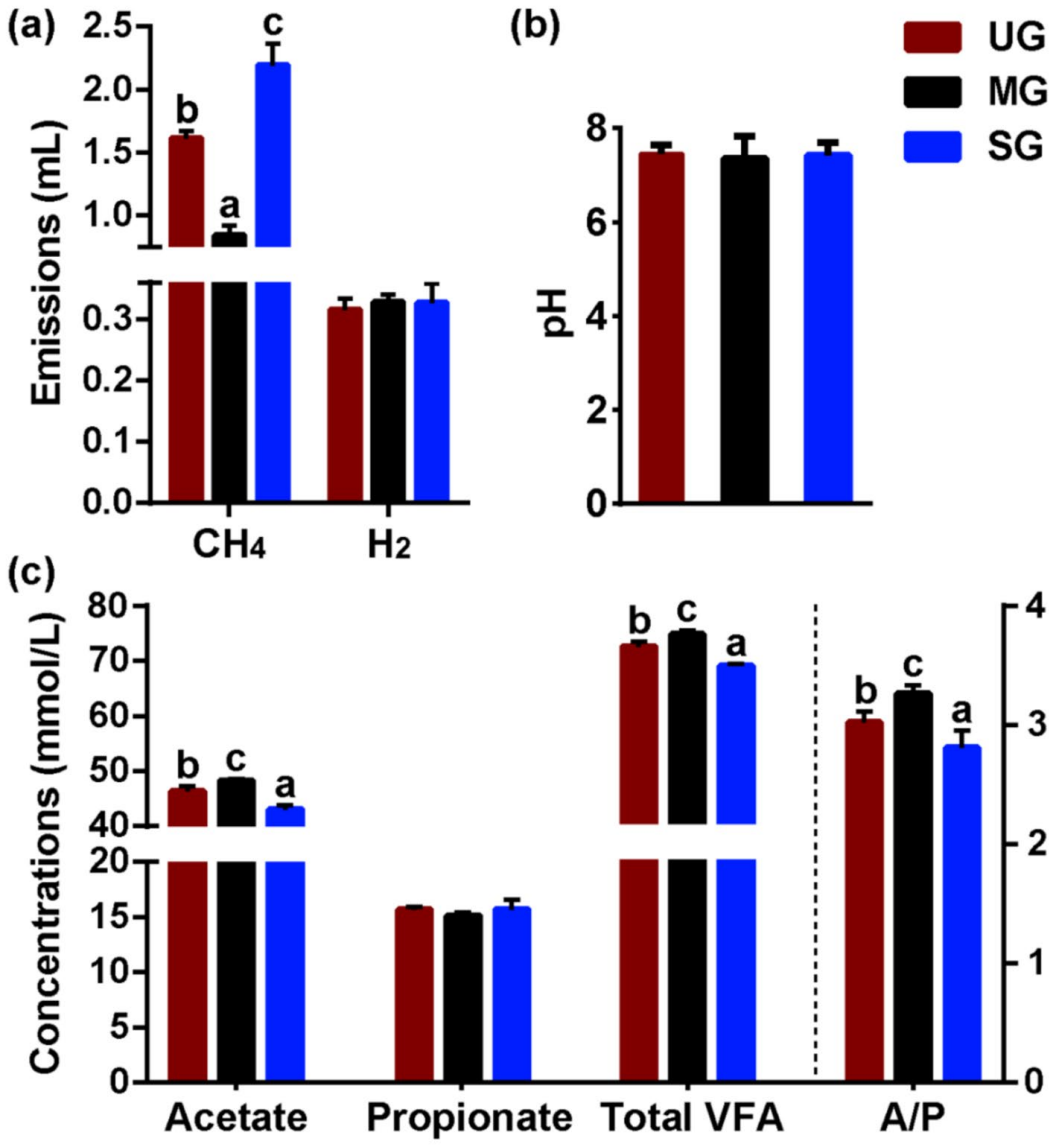
Rumen fermentation parameters in sheep fed diets simulating different levels of grassland saline-alkaline degradation. **(a)** methane (CH_4_) and hydrogen (H_2_) emissions *in vitro* rumen experiment; **(b,c)** pH and volatile fatty acids (VFA) profile *in vivo* feeding experiment; UG undegraded grassland, MG moderately degraded grassland, SG severely degraded grassland, A/P ratio of acetate to propionate; values with different letters indicate significant differences (*p* < 0.05).

The results (Figure 2a) for *in vitro* fermentation showed that the CH_4_ production in the SG group was higher than that of UG group (*p* < 0.05). Conversely, the sheep from MG group produced significantly less CH_4_ than that of UG group (*p* < 0.05; Figure 2a). However, no difference for H_2_ production was observed among the three groups (*p* > 0.10), indicating variation in carbon was driving differences. In addition, we further observed that ratio of acetate concentrations to propionate concentrations, ruminal total volatile fatty acids and acetate concentrations in the SG group was significantly decreased, but their levels significantly increased in MG group compared with the UG group (*p* < 0.01; Figure 2c). However, the pH (Figure 2b) and propionate concentration (Figure 2c) in the rumen were not altered among the three groups (*p* > 0.10). Similarly, we did not observe any differences in the average daily gain of sheep among the groups (Table 1).

**Table 1:** Average daily gain of sheep and nutrient level of diet.

Items	UG	MG	SG
ADG (g/d)	88.81 ± 1.95	89.46 ± 1.74	91.05 ± 2.20
Nutritional level (%)			
DM	92.38	92.22	91.74
CP	6.94	7.90	8.14
EE	1.69	2.23	2.59
OM	86.53	86.47	84.47
NDF	65.15	63.17	63.50
ADF	36.51	35.14	32.88
Dietary mineral content (g/kg)			
Ca	3.92	4.40	3.53
P	2.31	2.10	1.96
Na	2.69	7.51	19.50
K	11.05	11.51	11.90
Mg	1.37	1.62	1.90
S	1.80	1.76	1.95
Zn	17.19	19.48	18.35
Fe	149.20	230.76	310.15
Cu	4.35	5.28	5.49
Mn	32.45	42.64	56.77

ADG, average daily gain; DM, dry matter; CP, crude protein; EE, ether extract; OM, organic compounds; NDF, neutral detergent fiber; ADF, acid detergent fiber.

### 3.2 Variation in rumen microbial community

**Figure 3:**
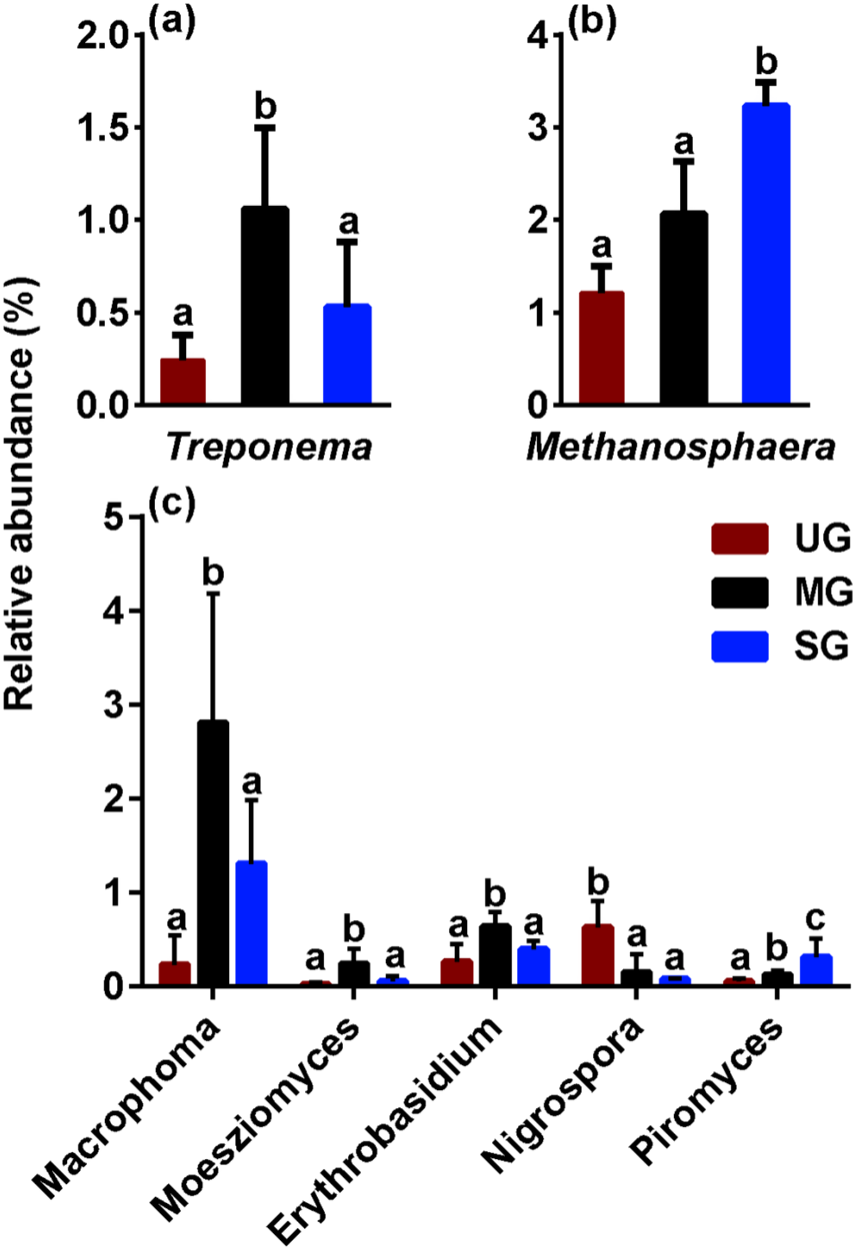
The relative abundance of key rumen bacterial **(a)**, archaeal **(b)**, and fungal **(c)** genus in sheep fed diets simulating different levels of grassland saline-alkaline degradation. UG undegraded grassland, MG moderately degraded grassland, SG severely degraded grassland; values with different letters indicate significant differences (*p* < 0.05).

We next focused particularly on the variation in microbes involved in the competition for carbon nutrition. Notably, we observed the relative abundance of *Treproema* that capable of converting carbon nutrition into acetic acid in the rumen of sheep in the MG group was significantly higher than that of sheep in the UG and SG groups (*p* < 0.05; Figure 3a). In addition, sheep in the SG group had higher ruminal *Methanosphaera*, which is a crucial role in the methane production by using carbon nutrition in relative abundance compared with the UG and MG groups (*p* < 0.05; Figure 3b). Among the different fungal genera, the relative abundance of *Macrophoma*, *Moesziomyces* and *Erythrobasidium* in the MG group increased significantly compared with the UG and SG groups, whereas the relative abundance of *Nigrospora* in sheep fed MG and SG diets decreased significantly compared with those sheep fed UG diet (*p* < 0.05; Figure 3c). Meanwhile, the *Piromyces* in relative abundance increased linearly with increasing levels of grassland saline-alkaline degradation (UG vs. MG vs. SG, all *p* < 0.05).

### 3.3 Correlation among various key microbes

**Figure 4:**
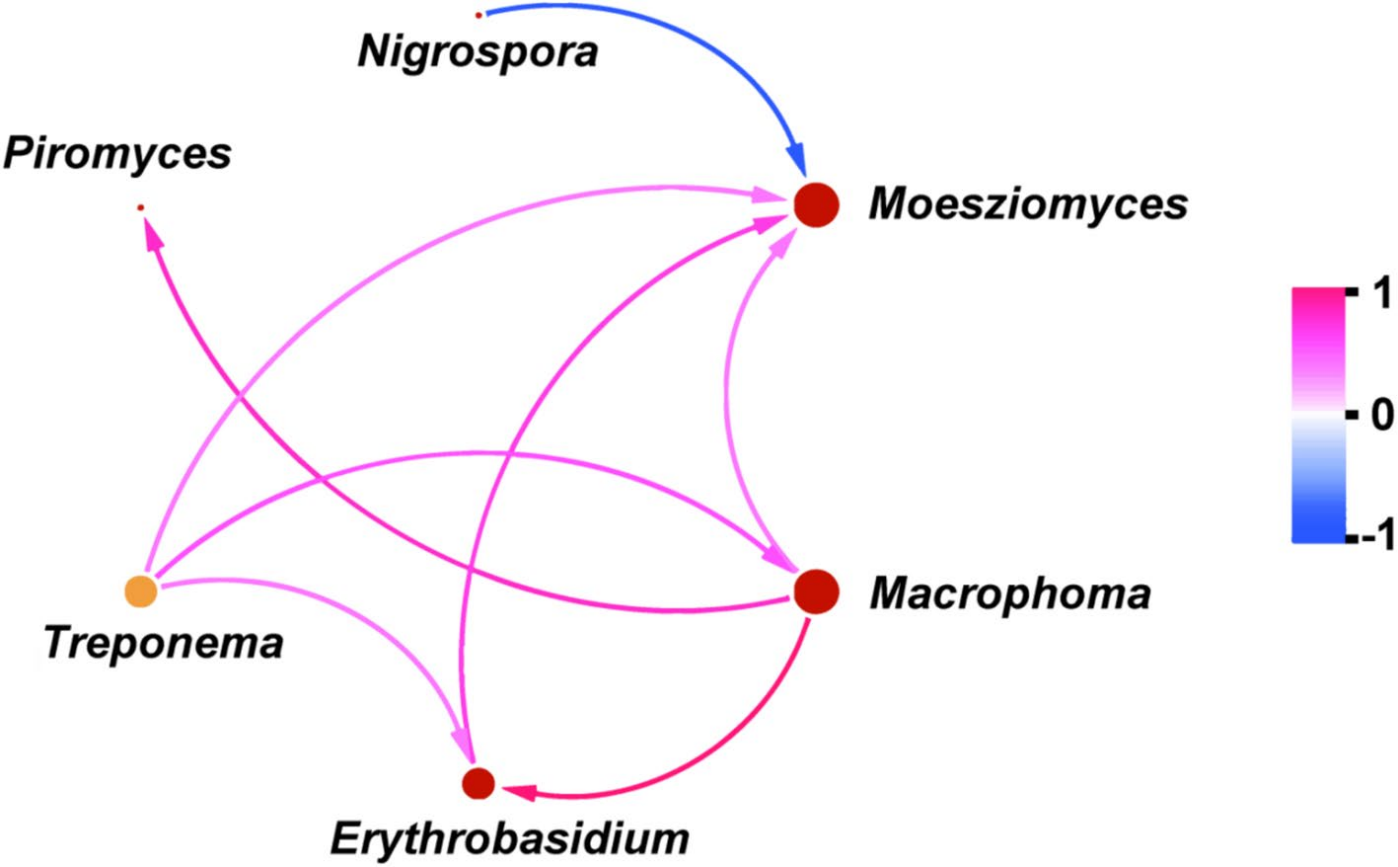
Analysis of the correlation among various key rumen microbes.

To investigate the underlying causes of the observed variation in carbon-nutrient competing microbes, we firstly conducted an analysis of the microbial network structure. Figure 4 showed that the *Moesziomyces* play the most important role among these key microbes, as it not only had a positive effect on *Macrophoma*, *Erythrobasidium* and *Treproema*, but also had a negative effect on *Nigrospora* (*p* < 0.05). Meanwhile, the *Macrophoma* and *Erythrobasidium* also had a positive effect on *Treproema* (*p* < 0.05). However, there was no significant effect of *Nigrospora* and *Erythrobasidium* on the *Treproema* (*p* > 0.10). Furthermore, our observations revealed that *Methanosphaera* was not integrated into the microbial network diagram, suggesting a potential insusceptibility of this genus to microbial interaction effects.

### 3.4 Nutrient intake

**Figure 5:**
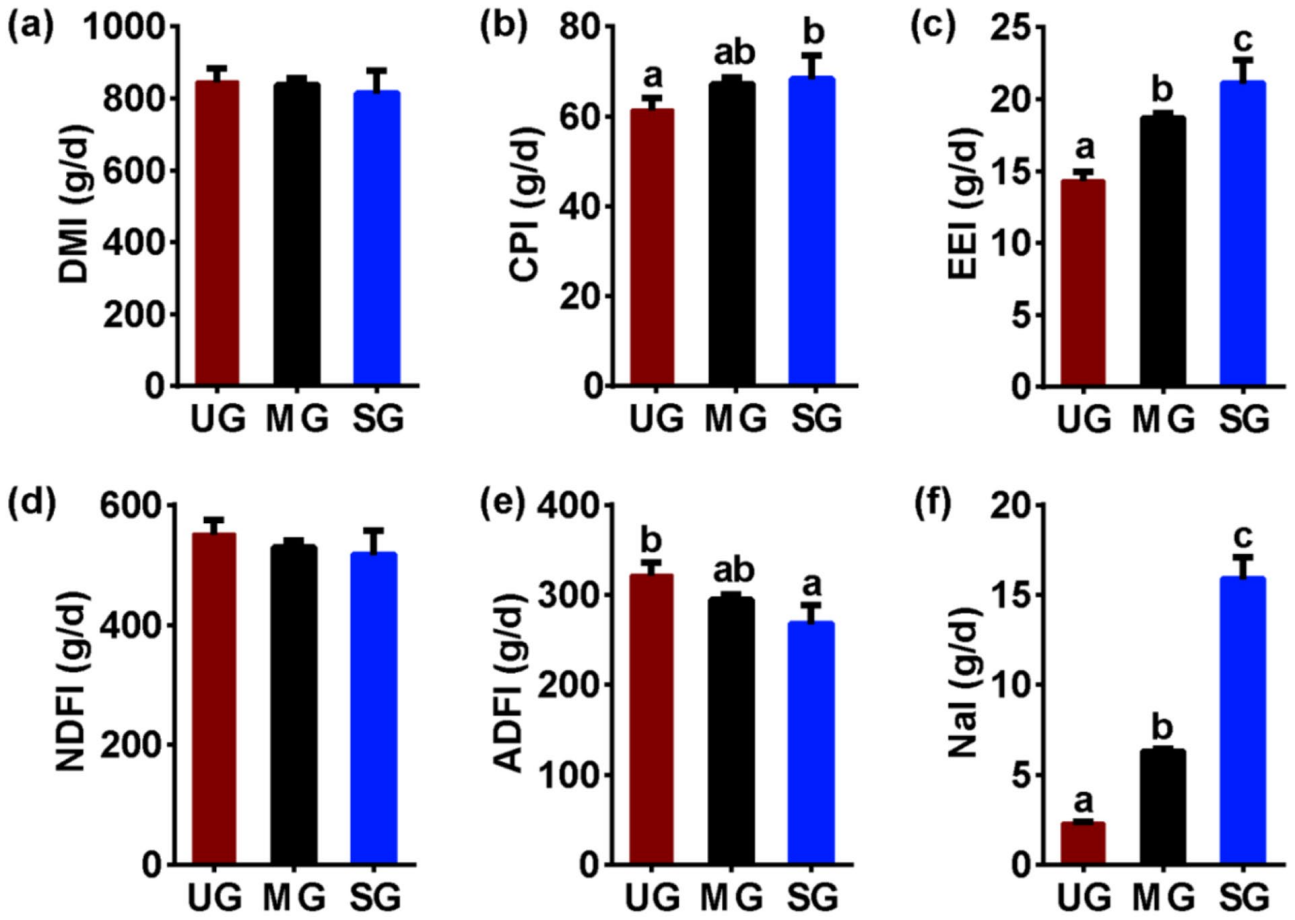
The nutrient intake in sheep fed diets simulating different levels of grassland saline-alkaline degradation. UG undegraded grassland, MG moderately degraded grassland, SG severely degraded grassland, DMI dry matter intake (Figure 5a), CPI, crude protein intake (Figure 5b), EEI ethyl ether extract intake (Figure 5c), NDFI neutral detergent fiber intake (Figure 5d), ADFI acid detergent fiber intake (Figure 5e), NaI Na intake (Figure 5f). values with different letters indicate significant differences (*p* < 0.05).

We further analyzed the significance of differences in nutrient intake among different groups of sheep. We found no significant differences in DM intake (Figure 5a)among the groups of sheep (*p* > 0.05), but the CP (Figure 5b) and EE (Figure 5c) intake of sheep in the SG group was higher (*p* < 0.05). Additionally, there were no significant differences in NDF intake (Figure 5d) among the groups of sheep (*p* > 0.05), while the ADF (Figure 5e) intake of sheep in the SG group was lower (*p* < 0.05). Notably, the Na intake (Figure 5f) of sheep increased linearly with the increasing levels of grassland saline-alkaline degradation (UG vs. MG vs. SG, all *p* < 0.05).

### 3.5 The effects of nutrient intake on *Methanosphaera* in the rumen of sheep

To identify the nutritional factors most likely to influence the variation in *Methanosphaera*, we next focused on analyzing the correlation between the nutritional resources consumed by those sheep with significant differences and *Methanosphaera* (Table 2). We found a significant effect of Na intake on *Methanosphaera* relative abundance (*p* < 0.01), but no significant effect of other nutrient intake on the *Methanosphaera* relative abundance was observed.

**Table 2:** Summary of linear mixed effects models analyzing the effects of nutrient intake to *Methanosphaera* relative abundance in the rumen.

Fixed factors	Methanosphaera			
Nutrient intake (g/d)	numDF	denDF	*F*-value	*p*-value
Na	1	7	40.99	<0.01
Ethyl ether extract	1	7	10.83	0.11
Crude protein	1	7	0.94	0.36
Acid detergent fiber	1	7	0.13	0.72

Na intake, ethyl ether extract intake, crude protein intake and acid detergent fiber intake were taken as fixed factors; grassland types (undegraded grassland plants, moderately degraded grassland plants and severely degraded grassland plants) were taken as random factors; numDF: the numerator degrees of freedom; denDF: denominator degrees of freedom.

## 4 Discussion

Our study is the first to evaluate the impact of grassland saline-alkaline degradation on herbivore CH_4_ emissions through feeding small-tailed sheep diets that simulated forage availability in grasslands with different levels of degradation. Our results found that the intake of moderately degraded grassland plants decreased CH_4_ emission in the rumen of sheep, however, the CH_4_ emission was higher in sheep that consumed severely degraded grassland plants (Figure 2a; UG: 1.61 mL vs. MG: 0.84 mL vs. SG: 2.19 mL). Moreover, we also further analyzed the rumen microbes, rumen fermentation parameters, and nutrient intake in sheep fed with different degraded grassland plants. These results provide a profound insight into how grassland saline-alkaline degradation affects CH_4_ emissions from sheep.

To our knowledge, the primary mechanism of the ruminal CH_4_ synthesis is the hydrogenotrophic pathway, which accounts for approximately 78% of total CH_4_ production (37). The abundance of methanogens and acetogenic bacteria are two important factors affecting this pathway in the rumen (10). In this pathway, methanogens can utilize H_2_ and CO_2_ to generate CH_4_, while acetogenic bacteria can also use the H_2_ to synthesize acetate (38). Thus, when the abundance of one of the microbe changes, the metabolic pathway of H_2_ shifts due to competition for substrates, which in turn affects CH_4_ production in the rumen. Our results showed that sheep fed diets simulating severely degraded grassland exhibited a notable increase in the abundance of *Methanosphaera* (Figure 3b), which has been proven to be a key methanogen in the rumen (13). Hence, the increased *Methanosphaera* in relative abundance may contribute to enhanced CH_4_ production via shifting the H_2_ toward methanogenesis pathway. Furthermore, our results also found that the relative abundance of *Treproema* in the rumen of sheep in the MG group was significantly higher than that of sheep in the UG and SG groups (Figure 3a). Further correlation analyses among various key microbes indicated that the increased relative abundance of *Moesziomyces* in the rumen of sheep fed moderately degraded grassland plants mainly driven the increasing in the relative abundance of *Treproema* (Figures 3c, 4). This phenomenon may be explained by the microbial food web theory proposed by Mizrahi et al. (39), who found that microbial communities undergo cascading metabolism in the rumen in a complex and coordinated manner, with continuous, cross-feeding relationships between different rumen microbes across the food web. Indeed, previous study demonstrated that *Moesziomyces* has the ability to degrade various plant polysaccharides by secreting plant cell wall-degrading enzymes (40). Although research on *Moesziomyces* in the ruminal environments remain limited to date, our findings suggest that this genus may contribute to provide more available nutrients for the *Treproema* to metabolize at the next trophic-like level. More importantly, previous research has recognized the *Treproema* is an acetogenic bacteria in the rumen, which is able to compete with methanogens for the utilization of H_2_ to produce acetate (41). Therefore, the increased *Treproema* in relative abundance may contribute to the decreased CH_4_ (Figure 2a) production and increased acetate concentration (Figure 3c) in the rumen of sheep fed moderately degraded grassland plants.

Evidence from experimental study has demonstrated that the rumen microbes were mainly affected by diet nutrient profiles (15), and therefore, grassland saline-alkaline degradation-induced alterations in plant resources in this study may be a key factor in causing an altered rumen microflora in sheep as described above, thereby further influencing CH_4_ emission. Our results found that as the level of grassland saline-alkaline degradation increased, the intake of ADF in sheep significantly decreased (Figure 5e), whereas the intake of EE significantly increased (Figure 5c). The decrease in ADF intake is often considered to reduce CH_4_ production in the rumen by reducing the abundance of fibrinolytic bacteria, which are major contributor for producing H_2_ for methanogens to synthesize CH_4_ (10, 42, 43). However, we found that the relative abundance of fibrinolytic bacteria (Figures 3a,c) and H_2_ production (Figure 2a) in the rumen of the SG group did not decrease. This observation may be attributed to the smaller changes in fiber intake between groups as compared to other studies (44, 45), suggesting that the alterations might not be substantial enough to induce variations in H_2_ content in the rumen. Moreover, although some fatty acids can reduce CH_4_ production by suppressing the number of methanogens and reducing H_2_ concentration through unsaturated bonds (21), the H_2_ content (Figure 2a) and abundance of methanogens (Figure 3b) also did not decrease with increasing EE intake in sheep. This phenomenon may be attributed to saline-alkaline grassland plants that lack specific fatty acids that reduce CH_4_ release from grazing animals. Consequently, we believe that grassland saline-alkaline degradation-induced alterations in ADF and EE intake was not the key factor affecting CH_4_ emissions in sheep. This opinion was further confirmed by the results of the linear mixed effects modeling analysis in this study, which found that there was no significant effect of ADF and EE intake on the relative abundance of *Methanosphaera* (Table 2).

**Figure 6:**
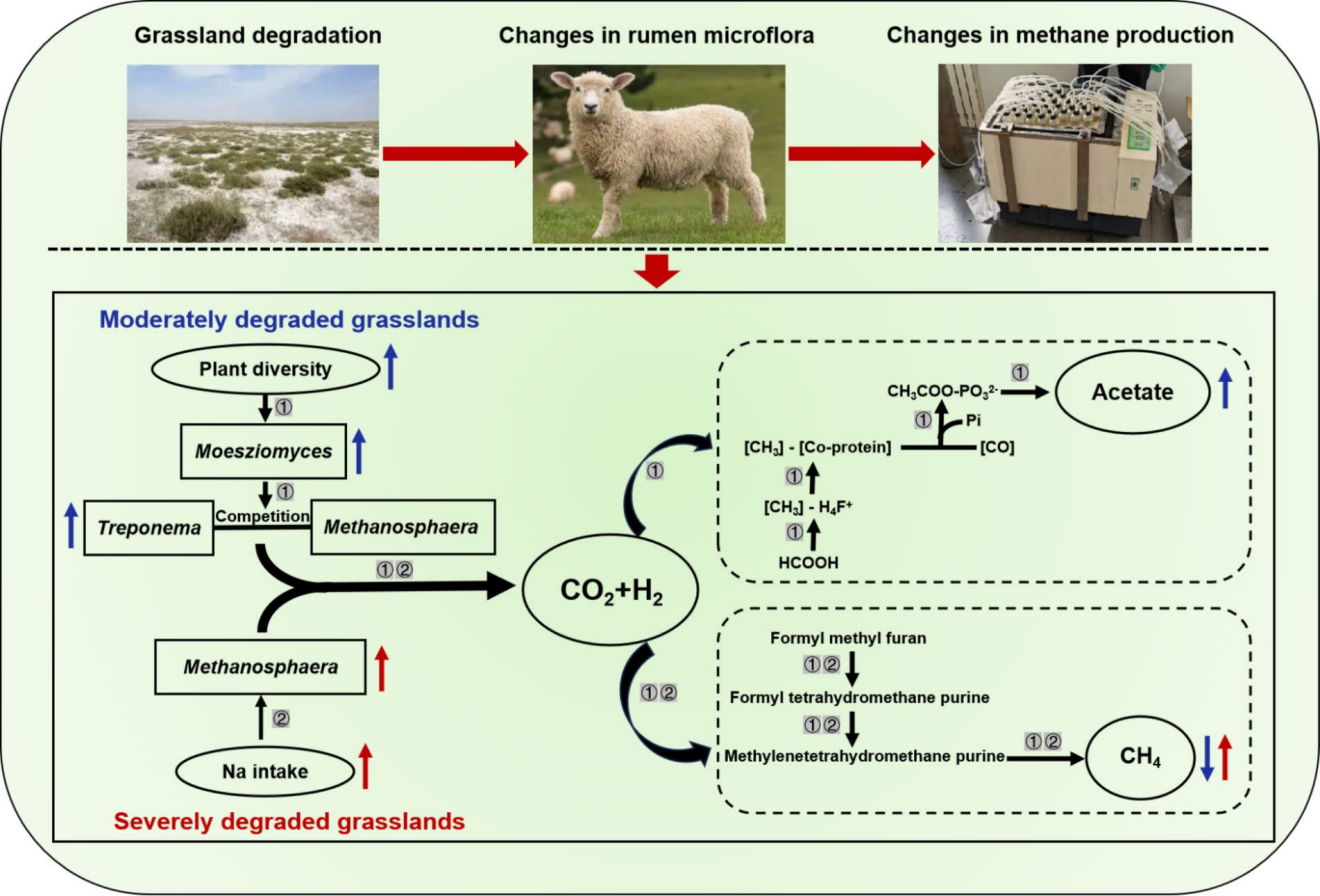
The underlying mechanisms of grassland saline-alkaline degradation affecting methane emissions of sheep. ① represents the pathway of influence of moderately degraded grasslands on methane emissions from sheep; ② represents the pathway of influence of severely degraded grasslands on methane emissions from sheep; 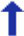
and 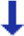 represents the related parameter of sheep fed diets simulating moderately degraded grasslands was higher and lower (*p* < 0.05) than that sheep fed diets simulating undegraded grasslands, respectively; 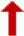 represents the related parameter of sheep fed diets simulating severely degraded grasslands was higher (*p* < 0.05) than that sheep fed diets simulating undegraded grasslands.

Interestingly, we are the first to find that excessive intake of Na might be the reason for increases in abundance of *Methanosphaera* (Table 2), thereby increasing the CH_4_ emissions of sheep fed severely degraded grassland plants. Despite previous studies have not focused on the association between Na intake and rumen methanogens, published research has found that methanogenic archaea primarily inhabited areas with higher concentrations of Na (46). Similarly, adenosine triphosphate synthase inhibitors can only decrease methanogenesis under low Na concentrations (47). These studies suggested that *Methanosphaera* may prefer saline environments. Therefore, an increase in Na intake may be a new pathway to promote the proliferation of methanogens for CH_4_ production. Our other important finding is that the plant species diversity and functional diversity in moderately degraded grassland was higher than that in undegraded and severely degraded grasslands (Figure 1). An interesting study demonstrated that the provision of a more diverse nutrient source (nine polysaccharides: starch, mucin, galactan, pectin, arabinogalactan, hemicellulose, cellulose, hyaluronan, and chondroitin sulfate) to microbes enables these species to coexist in high abundance by improving the mutual complementarity of their nutritional preferences (48). Hence, more diverse nutrient availability in moderately degraded grasslands, especially fiber, may contribute to increased relative abundance of *Moesziomyces*, which in turn may further increase the relative abundance of *Treproema*, thereby reducing the CH_4_ production via shifting the H_2_ toward acetate synthesis pathway. In general, our results suggested that changes in Na content and plant diversity induced by grassland saline-alkaline degradation may be the major contributors to influencing CH_4_ emissions of sheep by altering their rumen microbial community (Figure 6), which is worth further exploration.

## 5 Conclusion

Sheep that consume severely degraded grassland plants with excessive Na content experience an increase in the relative abundance of ruminal *Methanosphaera*. This shift leads to a redirection of H_2_ utilization toward CH_4_ production pathways. However, moderately degraded grassland plants increase the relative abundance of ruminal *Treproema* in sheep, which could inhibit the formation of CH_4_ via shifting the H_2_ toward acetate synthesis pathway. Overall, our results highlight that Na to be an important factor influencing ruminant CH_4_ emissions and also implicate that plant diversity as a possible another factor influencing CH_4_ emissions, but need to be further explored. Our study provides experimental evidence that livestock grazing on severely degraded saline-alkaline grasslands may exacerbate the adverse environmental effects of grassland degradation.

## Data Availability

The datasets presented in this study can be found in online repositories. The names of the repository/repositories and accession number(s) can be found in the article/Supplementary material.
