# Supplementary material for: Impact of grassland saline-alkaline degradation on domestic herbivore rumen microbiota and methane emissions
Source: Front Vet Sci. 2025 Jul 15;12:1598973. doi: 10.3389/fvets.2025.1598973 (PMC12303822; doi:10.3389/fvets.2025.1598973)
Supplement: Supplementary file 1 [file Table_1.docx]

SUPPLEMENTARY MATERIALS

**Supplementary Table 1** The nutrient intake in sheep fed diets simulating different levels of grassland saline-alkaline degradation. UG undegraded grassland, MG moderately degraded grassland, SG severely degraded grassland.

| Nutrient intake (g/d) | UG | MG | SG | *p*-value |
| --- | --- | --- | --- | --- |
| Na | 2.28 ± 0.05 | 6.29 ± 0.07 | 15.89 ± 0.61 | <0.01 |
| Ethyl ether extract | 14.32 ± 0.32 | 18.64 ± 0.20 | 21.11 ± 0.81 | <0.01 |
| Crude protein | 61.36 ± 1.39 | 67.21 ± 0.72 | 68.31 ± 2.62 | <0.05 |
| Acid detergent fiber | 321.69 ± 7.27 | 294.31 ± 3.17 | 267.88 ± 10.27 | <0.01 |

**The model formula of linear mixed effects models (LMMs)**

data1<-read.csv("GLM.csv",header = T)

library(nlme) #liner mixed mode

#install.packages("Tukey")

library(multcomp) #Tukey

library(sjstats)

#install.packages("sjstats")

library(lmerTest)

#install.packages("lmerTest")

install.packages("glmm.hp")

library(glmm.hp)

fit1<-lme(Methanosphaera~CP,random = ~ 1|grassland,data=data1)

anova(fit1)

summary(fit1)

r.squaredGLMM(fit1)
